# Supplementary material for: Crosscutting environmental risk with design: A multi-site, multi-city socioecological approach for Iowa’s diversifying small towns
Source: PLoS One. 2021 Jun 23;16(6):e0252127. doi: 10.1371/journal.pone.0252127 (PMC8221475; doi:10.1371/journal.pone.0252127)
Supplement: S1 File — (DOCX) [file pone.0252127.s001.docx]

S1 File. Appendices

Appendix 1: EPA EJscreen Environmental Indicators from [www.epa.gov/ejscreen](http://www.epa.gov/ejscreen)

| **Key \| Medium** | **Indicator** | **Details** | **Source** | **Date \| Year** | **National Average** |
| --- | --- | --- | --- | --- | --- |
| Air | National Scale Air Toxics Assessment (NATA) air toxic cancer risk | Lifetime cancer risk from inhalation of air toxics | EPA NATA | 2011 |  |
| Air | NATA respiratory hazard index | Air toxics respiratory hazard index (ratio of exposure concentration to health-based reference concentration) | EPA NATA | 2011 |  |
| Air | NATA diesel PM | Diesel particulate matter level in air, µg/m^3^ | EPA NATA | 2011 |  |
| Air | Particulate matter | PM2.5 levels in air, µg/m3 annual avg. | EPA, Office of Air and Radiation (OAR) fusion of model and monitor data | 2014 |  |
| Air | Ozone | Ozone summer seasonal avg. of daily maximum 8-hour concentration in air in parts per billion | EPA, OAR fusion of model and monitor data | 2014 |  |
| Air/ Other | Traffic proximity and volume | Count of vehicles (AADT, avg. annual daily traffic) at major roads within 500 meters, divided by distance in meters (not km) | [Calculated from 2014 U.S. Department of Transportation (DOT) traffic data, retrieved 2016](http://www.rita.dot.gov/bts/sites/rita.dot.gov.bts/files/publications/national_transportation_atlas_database/2014/index.html) | 2014 |  |
| Dust/ lead paint | Lead paint indicator | Percent of housing units built pre-1960, as indicator of potential lead paint exposure | Calculated based on Census/American Community Survey (ACS) data, retrieved 2018 | 2012-2016 |  |
| Waste/ air/ water | Proximity to Risk Management Plan (RMP) sites | Count of RMP (potential chemical accident management plan) facilities within 5 km (or nearest one beyond 5 km), each divided by distance in kilometers | Calculated from EPA RMP database, retrieved 05/09/2018 | 2018 |  |
| Waste/ air/ water | Proximity to Hazardous Waste Facilities | Count of hazardous waste facilities (TSDFs and LQGs) within 5 km (or nearest beyond 5 km), each divided by distance in kilometers | [TSDF data calculated from EPA RCRAInfo database, retrieved 06/11/2018](https://www3.epa.gov/enviro/facts/rcrainfo/search.html)  [LQG data calculated from the Biennial Hazardous Waste Report, retrieved 10/17/2017](https://www.epa.gov/hwgenerators/biennial-hazardous-waste-report) | 2018 |  |
| Waste/ air/ water | Proximity to National Priorities List (NPL) sites | Count of proposed or listed NPL - also known as superfund - sites within 5 km (or nearest one beyond 5 km), each divided by distance in kilometers | [Calculated from EPA CERCLIS database, retrieved 05/08/2018](http://cumulis.epa.gov/supercpad/cursites/srchsites.cfm) | 2018 |  |
| Water | Wastewater Dischargers Indicator (Stream Proximity and Toxic Concentration) | RSEI modeled Toxic Concentrations at stream segments within 500 meters, divided by distance in kilometers (km) | [Calculated from RSEI modeled toxic concentrations to stream reach segments, created 01/2017](https://www.epa.gov/rsei) | 2017 |  |

Appendix 2. Factor Loadings for Environmental Risk

The majority of risk factors positively loaded onto the first factor with RMP Proximity primarily loading onto the second. Follow up bi-variate correlations identify that PM 2.5 loads onto factor 1 (.71) and is significantly correlated with low-income populations (.53); NATA Diesel loads onto factor 1 (.471) and correlates with Demographic (.39), Minority (.33), less than high school (.44), and population under 5 (.51); NATA Air Toxics for Cancer Risk loads high (.889) and is correlated with Demographic (.43), Minority (.36), Low Income (.56), Less than H.S. (.55), and Under 5 (.55); Lead Paint also loads high (.778) and is correlated with less than high school (.37), under 5 (.35), and inversely with over 64 (-.56); last, RMP Proximity loads onto the two factors (.277, .878) respectively and is significantly correlated across populations with the exception of under 5 and inversely with over 64.


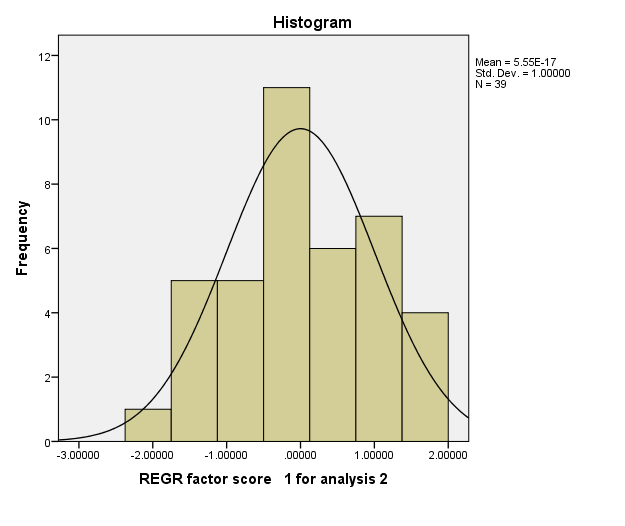


Appendix 3. Factor Loadings and Vulnerability

The PCA revealed two components: one with an eigenvalue of 3.52 that explained 58.7% of the total variance in the six variables; and a second one with an eigenvalue of 1.008 that explained 16.8% of the variance. Upon closer inspection, demographic communalities tend to be clustered by location with Factor 1 loading multiple indices of vulnerability (Table 4), such as minority population values (.842), linguistic isolation (.796), less than high school (.875), and population under age 5 (.675). While Factor 2 also loaded low income population (.621), less than high school (.275), and a population over 64 (.42). The components reflect the “parallel communities” often referred to in the literature between new and existing residents, with population age over 64 negatively correlating with more recent minority populations (r(39)=-.70, p<.001).


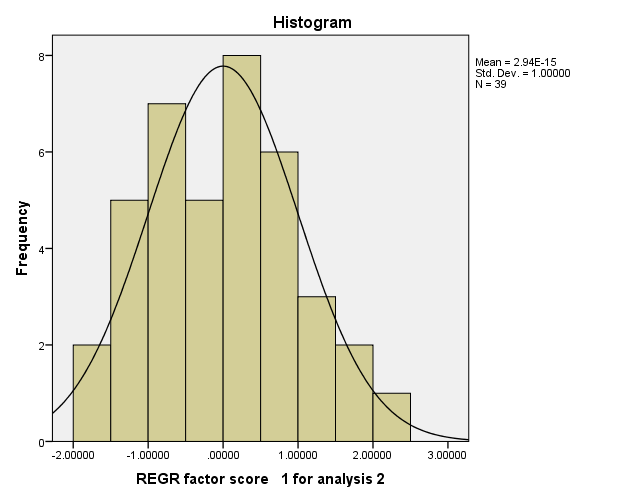


Appendix 4. Correlates of Environmental Risk and Vulnerability

Appendix 5. Fulcrum survey app showing how criteria was selected for each category.


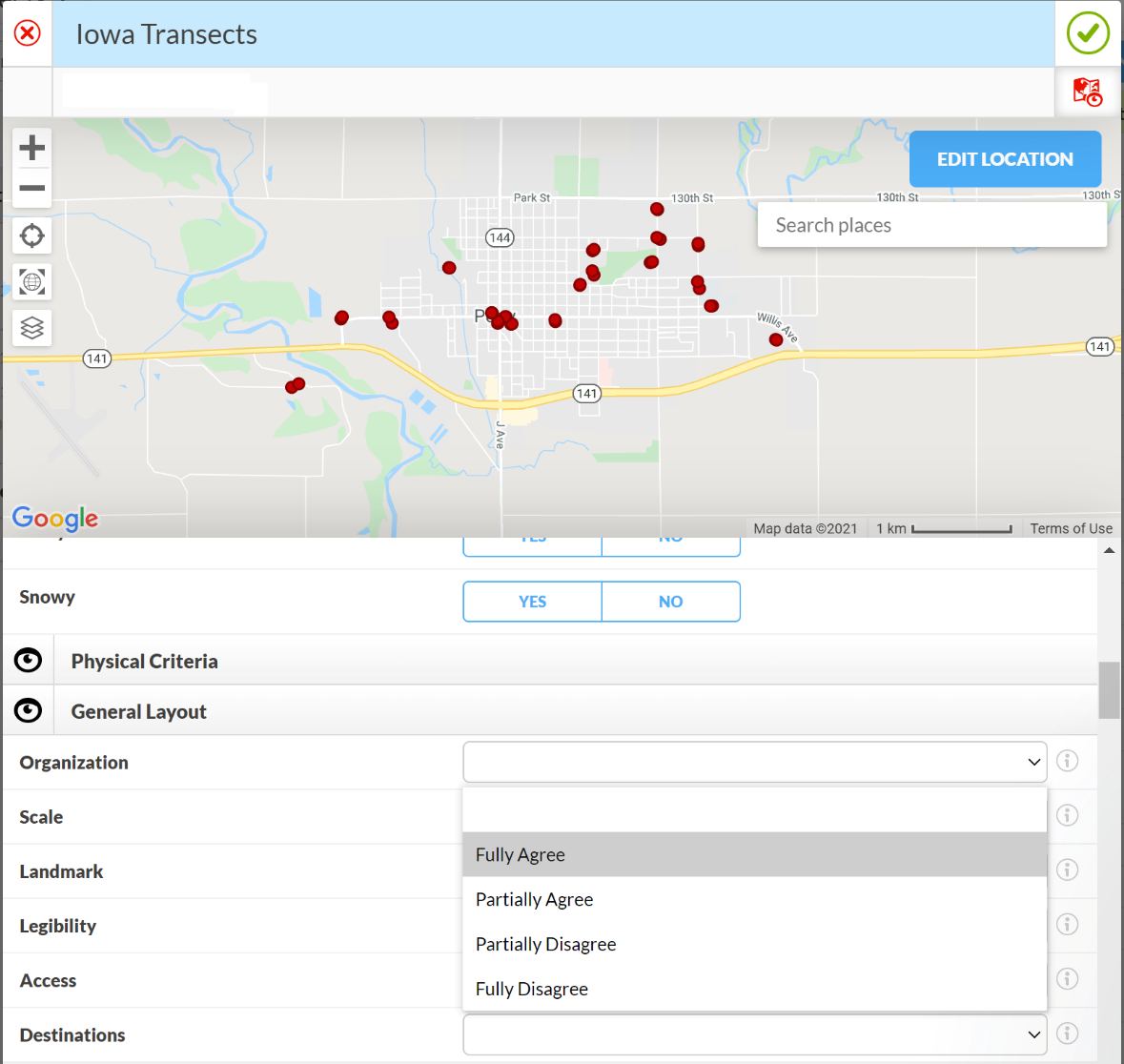


Appendix 6. Exploratory Matrix of Global Moran’s I of significant MLM POE coefficients with Environmental Risk (ER). Environmental design variables are dichotomous with lower y-axis scores indicating low access to environmental resources and lower x-axis score indicating lower environmental risk. Global Moran’s I using linear spatial autocorrelation to identify how variables cluster use spatially lagged transect points (latitude, longitude). The exploratory analysis support MLM models suggesting how environmental risk and environmental design are nested in transect points.

| 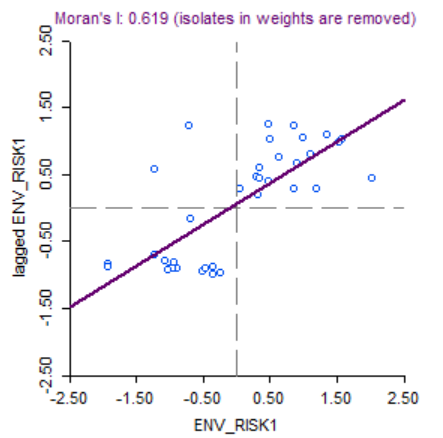 | 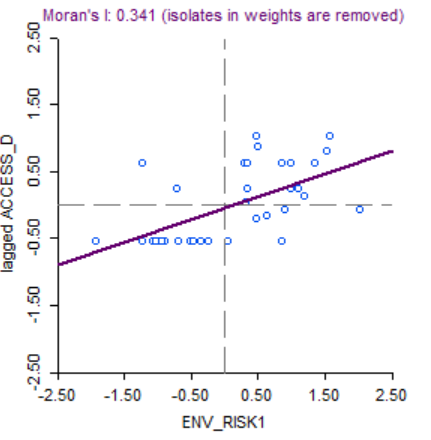 |
| --- | --- |
| 1. Environmental Risk (ER) clusters from low risk points to high risk left to right with Transect Points. ER is the horizontal axis throughout the matrix with left quadrants being low risk and right quadrants high. | 2. ER (x-axis) with Access (y-axis). High risk and high access influenced by proximity of diesel and traffic. |
| 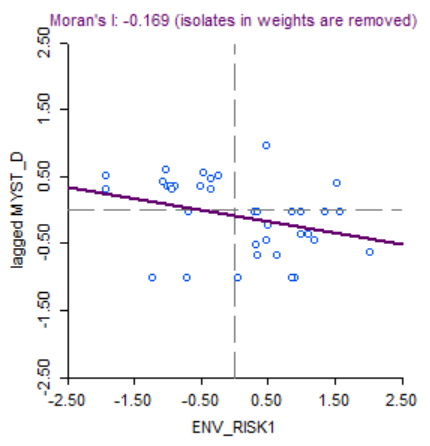 | 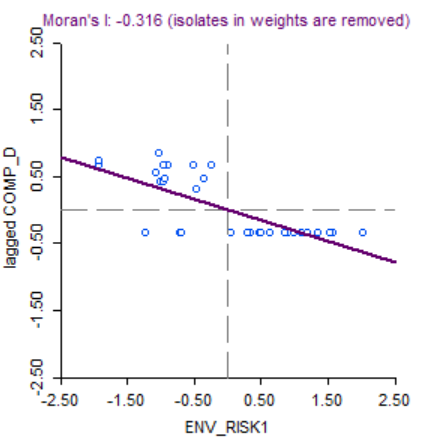 |
| 3. ER with Mystery. High mystery clusters with low risk and low mystery clusters with higher risk. | 4. ER with complexity. |
| 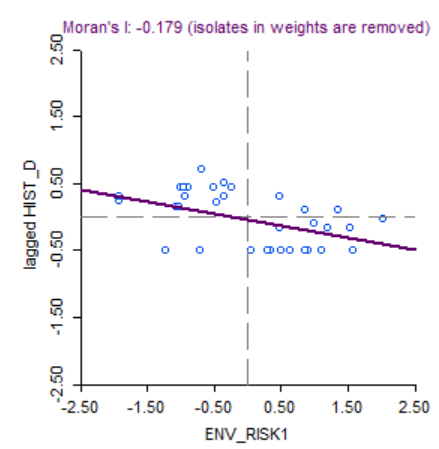 | 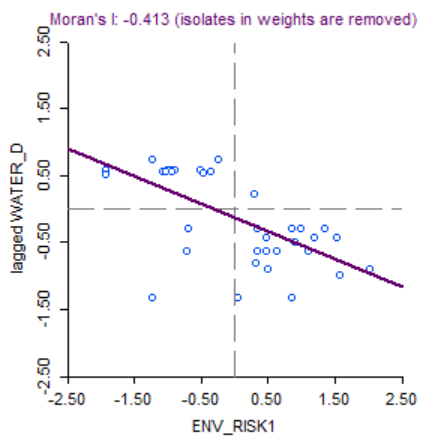 |
| 5. ER with History | 6. ER with Water |
| 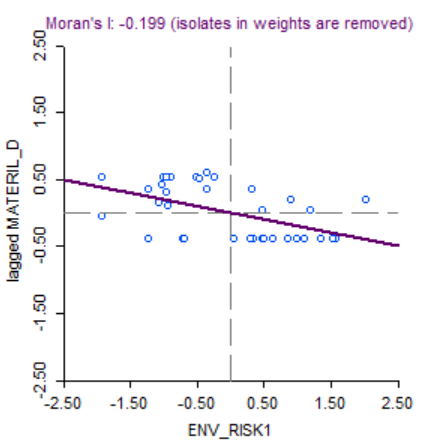 | 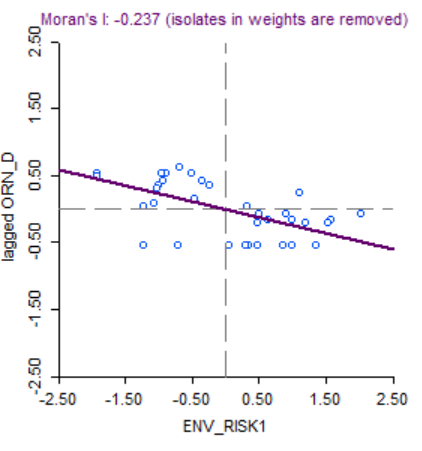 |
| 7. ER with Materiality | 8. ER with Big Trees |
| 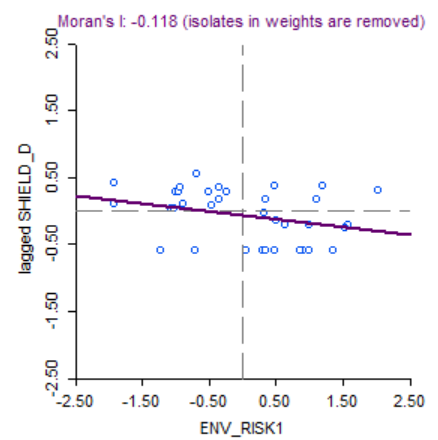 | 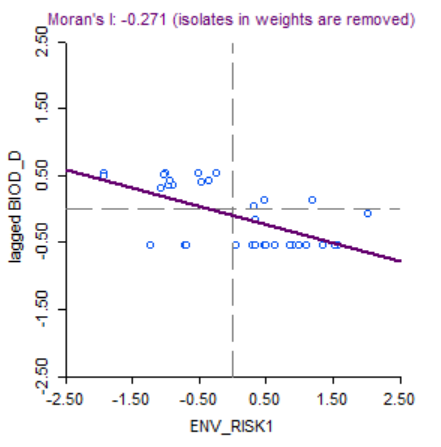 |
| 9. ER with Shield | 10. ER with Biodiversity |
| 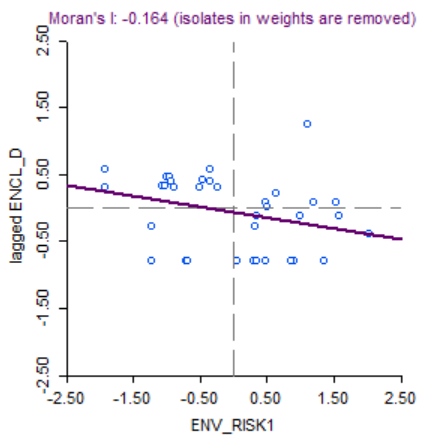 |  |
| 11. ER with Enclosure |  |
