# Supplementary material for: Crosscutting environmental risk with design: A multi-site, multi-city socioecological approach for Iowa’s diversifying small towns
Source: PLoS One. 2021 Jun 23;16(6):e0252127. doi: 10.1371/journal.pone.0252127 (PMC8221475; doi:10.1371/journal.pone.0252127)
Supplement: Attachment — Submitted filename: Response to Reviewers.docx [file pone.0252127.s003.docx]

Response to Reviewers

1. Please ensure that your manuscript meets PLOS ONE's style requirements, including those for file naming. The PLOS ONE style templates can be found at

<https://journals.plos.org/plosone/s/file?id=wjVg/PLOSOne_formatting_sample_main_body.pdf> and

<https://journals.plos.org/plosone/s/file?id=ba62/PLOSOne_formatting_sample_title_authors_affiliations.pdf>

2. We note that you have indicated that data from this study are available upon request. PLOS only allows data to be available upon request if there are legal or ethical restrictions on sharing data publicly. For more information on unacceptable data access restrictions, please see <http://journals.plos.org/plosone/s/data-availability#loc-unacceptable-data-access-restrictions>.

In your revised cover letter, please address the following prompts:

a) If there are ethical or legal restrictions on sharing a de-identified data set, please explain them in detail (e.g., data contain potentially sensitive information, data are owned by a third-party organization, etc.) and who has imposed them (e.g., an ethics committee). Please also provide contact information for a data access committee, ethics committee, or other institutional body to which data requests may be sent.

b) If there are no restrictions, please upload the minimal anonymized data set necessary to replicate your study findings as either Supporting Information files or to a stable, public repository and provide us with the relevant URLs, DOIs, or accession numbers. For a list of acceptable repositories, please see <http://journals.plos.org/plosone/s/data-availability#loc-recommended-repositories>.

We will update your Data Availability statement on your behalf to reflect the information you provide.

3. We note that Figure 1 in your submission contain map images which may be copyrighted. All PLOS content is published under the Creative Commons Attribution License (CC BY 4.0), which means that the manuscript, images, and Supporting Information files will be freely available online, and any third party is permitted to access, download, copy, distribute, and use these materials in any way, even commercially, with proper attribution. For these reasons, we cannot publish previously copyrighted maps or satellite images created using proprietary data, such as Google software (Google Maps, Street View, and Earth). For more information, see our copyright guidelines: <http://journals.plos.org/plosone/s/licenses-and-copyright>.

We require you to either (1) present written permission from the copyright holder to publish these figures specifically under the CC BY 4.0 license, or (2) remove the figures from your submission:

RESPONSE:

The base map roads are from the Iowa Department of Transportation and fall within the Creative Commons CCZero license of public domain data with a waiver of all rights including attribution. All other map material was created by the author.

3.1.    You may seek permission from the original copyright holder of Figure 1 to publish the content specifically under the CC BY 4.0 license.

We recommend that you contact the original copyright holder with the Content Permission Form (<http://journals.plos.org/plosone/s/file?id=7c09/content-permission-form.pdf>) and the following text:

“I request permission for the open-access journal PLOS ONE to publish XXX under the Creative Commons Attribution License (CCAL) CC BY 4.0 (<http://creativecommons.org/licenses/by/4.0/>). Please be aware that this license allows unrestricted use and distribution, even commercially, by third parties. Please reply and provide explicit written permission to publish XXX under a CC BY license and complete the attached form.”

Please upload the completed Content Permission Form or other proof of granted permissions as an "Other" file with your submission.

In the figure caption of the copyrighted figure, please include the following text: “Reprinted from [ref] under a CC BY license, with permission from [name of publisher], original copyright [original copyright year].”

RESPONSE: Added note to figure caption of CCZero license.

3.2.    If you are unable to obtain permission from the original copyright holder to publish these figures under the CC BY 4.0 license or if the copyright holder’s requirements are incompatible with the CC BY 4.0 license, please either i) remove the figure or ii) supply a replacement figure that complies with the CC BY 4.0 license. Please check copyright information on all replacement figures and update the figure caption with source information. If applicable, please specify in the figure caption text when a figure is similar but not identical to the original image and is therefore for illustrative purposes only.

The following resources for replacing copyrighted map figures may be helpful:

USGS National Map Viewer (public domain): <http://viewer.nationalmap.gov/viewer/>

The Gateway to Astronaut Photography of Earth (public domain): <http://eol.jsc.nasa.gov/sseop/clickmap/>

Maps at the CIA (public domain): <https://www.cia.gov/library/publications/the-world-factbook/index.html> and <https://www.cia.gov/library/publications/cia-maps-publications/index.html>

NASA Earth Observatory (public domain): <http://earthobservatory.nasa.gov/>

Landsat: <http://landsat.visibleearth.nasa.gov/>

USGS EROS (Earth Resources Observatory and Science (EROS) Center) (public domain): [http://eros.usgs.gov/#](http://eros.usgs.gov/)

Natural Earth (public domain): <http://www.naturalearthdata.com/>

[Note: HTML markup is below. Please do not edit.]

Reviewers' comments:

Reviewer's Responses to Questions

**Comments to the Author**

1. Is the manuscript technically sound, and do the data support the conclusions?

The manuscript must describe a technically sound piece of scientific research with data that supports the conclusions. Experiments must have been conducted rigorously, with appropriate controls, replication, and sample sizes. The conclusions must be drawn appropriately based on the data presented.

Reviewer #1: Yes

Reviewer #2: Partly

2. Has the statistical analysis been performed appropriately and rigorously?

Reviewer #1: Yes

Reviewer #2: No

3. Have the authors made all data underlying the findings in their manuscript fully available?

The [PLOS Data policy](http://www.plosone.org/static/policies.action#sharing) requires authors to make all data underlying the findings described in their manuscript fully available without restriction, with rare exception (please refer to the Data Availability Statement in the manuscript PDF file). The data should be provided as part of the manuscript or its supporting information, or deposited to a public repository. For example, in addition to summary statistics, the data points behind means, medians and variance measures should be available. If there are restrictions on publicly sharing data—e.g. participant privacy or use of data from a third party—those must be specified.

Reviewer #1: Yes

Reviewer #2: No

4. Is the manuscript presented in an intelligible fashion and written in standard English?

PLOS ONE does not copyedit accepted manuscripts, so the language in submitted articles must be clear, correct, and unambiguous. Any typographical or grammatical errors should be corrected at revision, so please note any specific errors here.

Reviewer #1: Yes

Reviewer #2: No

5. Review Comments to the Author

Please use the space provided to explain your answers to the questions above. You may also include additional comments for the author, including concerns about dual publication, research ethics, or publication ethics. (Please upload your review as an attachment if it exceeds 20,000 characters)

Reviewer #1: This study involves an interesting analysis of the built environment and correlates with well-being, specifically in rural areas with changing demographics. Analysis of the data appears sound overall, and the paper is generally well written. I have only a few comments and suggestions to improve the clarity of the presentation.

The term “ground-truthing” may be a bit misleading. The study uses both data derived from existing geospatial data sets and on-the-ground observations, but there is no verification of the geospatial data using the observations, as ground-truthing would imply.

Good suggestion. It would be inappropriate to suggest that geospatial environmental risk data could be “ground truthed” to predict poor POE. The objective was to see if there was a double-threat of environmental risk and poor, built environmental design. Title changed to reflect the intent of the paper: “Ground-truthing environmental risk with design”. Also added clarification of ground truthing in the methods section to due with visible verification of existing conditions.

I am somewhat familiar with POE for buildings, not for outdoor spaces. If this is an adaptation, or novel aspect of this work, please explain.

Revised the methods section to clarify that the POE used an outdoor, residential tool developed by researchers in Sweden.

By line 295 (and Table 3), it became apparent that the comparison is mainly between small towns and state averages, and not between rural areas and diversifying rural areas. Some of the introduction and background (and the title of the paper) led me to expect an analysis of the effects of diversification, but I suppose that would require a longitudinal (long-term) study, or a broader study of small towns including some that have diversified and others that have not.

This is an excellent point. State averages in the EPA data reflect urban environments because the majority of the population is urban, whereas rural areas remain outliers because they don’t have the density. I revised this section to note that state averages were used as a proxy for urban populations, noting that rural communities deviate substantially. Also, a comparison study of small towns that have diversified and those that have not is an excellent idea! I’ve added that to the limitations. I hope the inclusion of these revisions meets the reviewer’s expectations.

Specific Comments:

Line 50: Spell out SES the first time used. Addressed.

Line 64: Briefly define “parallel lives.” Addressed in lines 49 – 52.

Line 130: It’s not clear if the study is considering actual physical barriers (highways, railways, waterways) that can promote segregation of populations. Added the sentence: As suggested by Talen [36], the present study included natural and infrastructural barriers, like rivers and highways, known to isolate and segregate communities (see transects).

Lines 370-374: A figure (bar chart) or table might be helpful for presenting these results. Environmental risk and population vulnerability are now figures as bar charts. The visual comparison of the divergence between the two factors helps to tell the story, thanks for the suggestion.

Lines 398-400: It may be helpful to define some of these variables, unless they are well known in the field of landscape architecture. Good suggestion but it might be a challenge to respond within word limits. The concepts are well known in the landscape architecture community. To help I included a reference here to the manuscripts by Cross and Kuller that go into detail on what each of these concepts mean.

Reviewer #2: This manuscript intended to study the equity of environmental exposure and its built environment factors of the exposure in three small towns in Iowa. Specifically, the authors aimed to compare the exposure between small towns and general urban populations. To evaluate environmental exposure, the secondary EPA data were collected and compared with a state average. Then a post-occupancy evaluation was conducted to explore built environment characteristics. This is an interesting topic and it’s good to see this counterbalance aside from a myriad of urban studies on this topic. However, this manuscript is not ready to be published due to the fact that the analysis and rigorous in writing are not of sufficient quality. So I recommend major revision for this manuscript. My main concerns are listed below. RESPONSE: noted the myriad of urban studies papers on this topic. The original contribution from this research is its focus on small towns, which remain relatively understudied in this area.
1. The comparison between small towns and the state average is biased (Table 3), because the transect points are selective and supposed to have higher exposure to pollutants compared to the average in town – “each transect line had to cross environmental criteria potentially exacerbating risk, such as agricultural fields next to a public middle school” (line 229) RESPONSE: The first paragraph of the methods section acknowledges this approach, “The post-occupancy evaluation of study sites selected locations using a systematic sampling interval based upon variations in socio-ecological criteria, e.g. low-income housing in floodplains, through transects.” The approach overcomes known political ecology constraints, like red-lining or census tracts, that have either negatively impacted communities through segregation or don’t represent communities due to low-density. In response to the reviewer comment, I have added sampling bias to the limitations section to further acknowledge the approach.

2. The aim of this research currently stands is blurry given to the chosen terminology “environmental barrier”. In the literature in environmental health this term is often referred to the barrier for disabled people, or barriers to outdoor activities. However, this study is focusing on “exposure to environmental pollutants”. Plus the point about environmental exposure is also not clear in the introduction. It is highly recommended to make the research goal clearer and coherent throughout the title, abstract, as well as the introduction. RESPONSE: This is a really important comment and we appreciate the feedback. Please see paper for full extent of revisions, as the comment demanded a major revision. A summary of the changes follows: the title has been changed to emphasize the relationship between environmental risk and design with similar changes throughout the paper; environmental barrier removed and changed to environmental exposure and environmental design to be more specific. The abstract was revised substantially based upon this feedback. Hopefully, the objective is clearer and the use of POE to measure environmental support for behaviors is clearer.
(1) in the abstract, the aim of study is described in a very general way: “We aimed to understand the impact of built environments—all the physical parts of where people live and work—to contribute to healthy behaviors for vulnerable populations.” Please be specific on which part of the built-environment. RESPONSE: changed physical parts to outdoor environments. This eliminates vagueness related to housing, workplaces, and other indoor or otherwise inaccessible locations for survey research.

I am also wondering that did the authors include any variables of “healthy behaviors” in this work? RESPONSE: Added a sentence that healthy behaviors were not measured during the course of the study and emphasized environmental design to support healthy behaviors. Due to funding limitations, the survey portion of the research was eliminated, so no human subjects research was conducted. The POE and MLM strategy serves as a proxy to examine the extent to which the built environment supports activities related to healthy behaviors, which is a common approach in ground truthing research methods.

Why is environmental exposure missing in this objective? RESPONSE: revised the abstract to clearly indicate the study is looking at the correlation of environmental risk and design.

(2) In section 1.3 research question, the single hypothesis is that the environmental exposure in the small towns is not different from the exposure that general urban populations hold. Here, the role of built environment is missing in the hypothesis, which is conflicting to point (1). RESPONSE: removed “socio- and natural ecosystem services” and replaced it with supportive environmental design.
(3) following the previous point, if the equality of environmental exposure is the only research question, table 3 will be the only relevant result. Why POE analysis, PCA and MLM analysis are needed? RESPONSE: see notes throughout. This point has helped bring clarity throughout the paper.
3. It is not justified the design of multilevel model. If the class here is the towns, there would be only three groups. The suggested group size for multilevel model is 50 (Moineddin 2007). Otherwise, for a sample size of 39, a simple map could be clear enough to see the variation between transect locations if that is the only purpose to use multilevel model. RESPONSE: Excellent point although it might be difficult to map 206 variables across 39 locations or to statistically demonstrate significant differences. As noted, MLM has traditionally been used for students in classrooms in schools, but needing to achieve a threshold of 50 is a new constraint and one that I am not familiar. Neither Tabachnick (2007) or Hoffman (2007) suggest a group size of 50 as a threshold. Theall et al., referenced in this paper, found MLM produces reliable statistical analyses with group sizes <5. In justification of the method for the paper, the ICC and Betas from the MLM (Figure 5) demonstrate the environmental design specific to locations with supportive and non-supportive environments exist in the nested transect points across small towns. Parallel environments exist and they correlate with environmental risk. I added a better description of the MLM process and why this particular statistic was used because the study was designed to work with this statistical approach.

Here are some minor comments.
1. Replace the comma by a full stop in the end of the abstract. Revised.
2. Line 59 it’s unclear that if Ozkan is a kind of evaluation or an author name of the cited work until checking the reference. Added date.
3. Line 115 what does the combination “[emphasis added] (2008)” mean? Changed emphasis to italics.
4. Please revisit some sentences and paragraphs. For instance,
(1) can you put the concrete meaning of environmental barriers in line 129 to the first glance around line 52? Revised.
(2) Lines 144-146 the authors argue that traditional built-environment metrics such as density are not applicable to rural environments. Right after, can you state what BE components you use for this study instead? Revised.
(3) Line 202 “The built environment in small towns may serve parallel populations in a dichotomous manner with underrepresented minorities encountering a disproportionate access to environmental harms and benefits” and following with the hypothesis “The hypothesis, …, is that vulnerable residents at the intersection of low-SES and minority status are just as likely as wealthier, educated, English-speaking residents to live in environments lacking supportive socio- and natural ecosystem services and that no difference would be found…”. What is the connection between these two sentences? Why the provided context in the first sentence drives such an opposite hypothesis? Would you consider rephrase? Entire paragraph revised to make this clearer. Thank you for the suggestion.
(4) Line 212 methods, it is not clear that what the (n=39) sample size means without reading the text afterward. Does it mean towns? Residents? Transects? Revised.
(5) Line 267 how those secondary station data fits into transect points? What's the interpolation approach? How many points of observations were calculated around the study sites? This section was confusing and has been revised to be clearer.
5. At the paragraph lines 136-151, the authors did not mention components of the social environment, making the social criteria in Table 1 come at a surprise. Revised.
6. Please check if the POE variables in Table 6 and Table 2 are consistent. Can you put the definition of each variable in method (e.g. unique and history sound very fuzzy)? Table 6 uses some of the variables from table 2 but only the ones that were significant. The other reviewer also requested these terms to be defined but it would greatly expand the length of this already lengthy paper to explain. The reference to Cross and Kuller who created the scale and have published twice on the items has been emphasized.
7. The authors need to describe the population characteristics of those who conducted POE and discuss the limitation when generalize their perceptions. Added description of the graduate students with extensive training in design disciplines to be fitting of a POE.
8. The data sources of lead pain index and RMP facilities are missing. Removed text from document and redirected to appendices which provide a thorough list of sources.

General response to reviewer two. Thank you for your thorough feedback. The paper clearly suffered from too much jargon and we’ve thoroughly vetted the paper seeking to add clarity. Probably the most important point was that socio- ecosystem resources didn’t appear in the hypothesis as what was measured. The revised paper eliminates this over-complicated phrasing and focusing on environmental design to make it clear that POE makes sense.

6. PLOS authors have the option to publish the peer review history of their article ([what does this mean?](https://journals.plos.org/plosone/s/editorial-and-peer-review-process#loc-peer-review-history)). If published, this will include your full peer review and any attached files.


If you choose “no”, your identity will remain anonymous but your review may still be made public.


**Do you want your identity to be public for this peer review?** For information about this choice, including consent withdrawal, please see our [Privacy Policy](https://www.plos.org/privacy-policy).

Reviewer #1: No

Reviewer #2: No

[NOTE: If reviewer comments were submitted as an attachment file, they will be attached to this email and accessible via the submission site. Please log into your account, locate the manuscript record, and check for the action link "View Attachments". If this link does not appear, there are no attachment files.]

While revising your submission, please upload your figure files to the Preflight Analysis and Conversion Engine (PACE) digital diagnostic tool, <https://pacev2.apexcovantage.com/>. PACE helps ensure that figures meet PLOS requirements. To use PACE, you must first register as a user. Registration is free. Then, login and navigate to the UPLOAD tab, where you will find detailed instructions on how to use the tool. If you encounter any issues or have any questions when using PACE, please email PLOS at [figures@plos.org](mailto:figures@plos.org). Please note that Supporting Information files do not need this step.
