# Supplementary material for: Crosscutting environmental risk with design: A multi-site, multi-city socioecological approach for Iowa’s diversifying small towns
Source: PLoS One. 2021 Jun 23;16(6):e0252127. doi: 10.1371/journal.pone.0252127 (PMC8221475; doi:10.1371/journal.pone.0252127)
Supplement: Attachment — Submitted filename: Reviewer 3.docx [file pone.0252127.s004.docx]

This study aims to analyse the correlation between environmental risk and built environmental design and conducts precise analyses to demonstrate how the environment risks correlated with the built environment and vulnerability. The paper is well-organised, but the main worries are about the representation of environment design.

First, since the authors clarify that the aim is to see if there was a double threat of environmental risk and poor built environmental design, but the current presentation about environment design is still weak. The authors talked more about environment design and society (health, justices), how to measure environment design, how to evaluate environmental risks in the literature review, but the relationship between design and environmental risks is less mentioned. For example, in existing findings, what kinds of characteristics of the built environment can exacerbate the environmental risks, and can POE evaluate these characteristics effectively, is it validate to apply POE to study risk-related environment design? Or else, how to explain the usage of variables in table 6?

Second, although the analyses from 3.2, 3.3 and 3.4 are helpful, the necessary explanations of variables in MLM are in a lack, and the results are less convincing, which need more evidence. For example, ¨suggesting that places without easy access by car, bus, walking, or biking have reduced environmental risk¨, which is biased. If people cannot walk and cycle to these places, what is a need to study these places? The demonstrations about the analysis and results of MLM need improvement.

Additionally, more discussions about the results of MLM are in need. For example, why and how the variables are related to environmental risk from the findings of this paper. In all, the presentation about environmental risk and design should be investigated more.

**Minor comments:**

1) In the section of 2.5, the logic of analysis should be clarified, like the connections among PCA and MLM analyses.

2) Please pay attention to the length of the introduction and literature. The first two paragraphs in literature are repeatable with the introduction, it is better to make them concise.

3) Line 169-170, is this paper the first one to apply the instrument Cross and Küller? If not, it is good to mention how others use it?

4) Line 339-340, to be clear about the vulnerability, is it about healthy vulnerability or social vulnerability?

5) Line 389, the subtitle ¨Physical and social condition¨, which is not clear, is physical health or physical environment, society or socioeconomic factors?

6) Complement more information about the data (e.g.: year of data, quality).
