# Supplementary material for: Crosscutting environmental risk with design: A multi-site, multi-city socioecological approach for Iowa’s diversifying small towns
Source: PLoS One. 2021 Jun 23;16(6):e0252127. doi: 10.1371/journal.pone.0252127 (PMC8221475; doi:10.1371/journal.pone.0252127)
Supplement: Attachment — Submitted filename: Review 3 Rebuttal.docx [file pone.0252127.s005.docx]

Review 3 Rebuttal

6. Review Comments to the Author

Please use the space provided to explain your answers to the questions above. You may also include additional comments for the author, including concerns about dual publication, research ethics, or publication ethics. (Please upload your review as an attachment if it exceeds 20,000 characters)

Reviewer #3: This manuscript presented a series of analyses including post occupancy evaluation (POE), principal component analysis (PCA), and multi-level model (MLM) to study the parallel communities in several small towns in Iowa, for the understanding of how built environment affect the quality of living and health risks to different people. This is the second time I review this manuscript, and all my previous concerns were addressed in current version. My new concerns are about the MLM analysis and interpretation which I couldn't review for, due to the incompleteness of previous version. There are also some minor issues that require further refine before publication.

1. Figure 2, the Y-axis and legend are missing. I can imagine blue bars are component 1 and orange bars are component 2, but please be explicit as this is a formal publication. In addition, the information in Figure 2 and Table 4 is exactly same, hence redundant.

Thanks for the recommendation and catching this oversight. Y-axes are now labeled, legend corrected to reflect the statistical measure, and tables 3 and 5 have been removed to decrease redundancy.

2. Same as above for Figure 3. And Figure 3/Table 5.3. For Figure 4, the estimated ‘beta’(s) are known as coefficient(s) or slopes, but the term coefficient is more preferred; and the main issue is that the term ‘beta’ is less formal and not preferred.

Coefficients are used whenever possible. However, we differentiate when necessary between intercepts and slopes. We no longer refer to betas as we agree with the reviewer that the term may be too colloquial and may not be appropriate given that the coefficients are not standardized.

Corrected on caption and in text body.

4. Second issue about Figure 4 is that in the figure, the Y-axis is written as ‘Increase in Environmental Risk’—how does this MLM slopes can be used to interpret as the ‘increase in environmental risk’? Please explain.
This is an excellent point which brings up a larger set of concerns about the figure regarding interpretability. We have consulted with a MLM expert to ensure that our interpretation and presentation are accurate. The figure and its legend has been completely re-done to enhance clarity. We have edited the Y-axis to indicate that the Y-axis is a PCA score of environmental risk and we clarify in the legend that the PCA scale is standardized such that a score of 1 is one standard deviation above the mean.

Next, the reviewer was correct in pointing out that the conflation of intercepts and slopes in the figure was misleading. Therefore, we have converted all of the coefficients from slopes into predicted scores for sites that have each form of POE. Doing so allows the intercept to be interpreted as the expected score for sites that do not have each POE and the ‘slope’ to be the predicted score for sites that do have each POE.

We also agree with the reviewer that the figure suggested that the respective POEs could be compared, but this is not the case and we clarify in the figure caption that the individual POEs can be compared in terms of meeting or lacking an urban item, but readers should refrain from comparisons across POEs. We considered separating out the figure into separate graphics, and opted to add exploratory Global Moran’s I clusters of significant MLM and POE effects to the supplement in appendix 6. We have also added observed frequency information to the figure so that the reader can contextualize the magnitude of the POEs. That is, some POEs such as complexity are high impact but relatively rarely observed and thus may exert less of a population level impact.

5. The process of running the MLM is not clear. E.g., what is(are) the dependent variable(s) in the ‘multiple multilinear models’ (page 18 line 391)? How does the ‘multiple multilinear model’ works? This line (391) is under section of ‘3.5 POE and MLM’, but no ‘multilinear model’ is described in analysis method section (sec 2.5). Based on the sec 2.5 lines 293-306, as a reader I would expect only one multivariate multilevel model will be presented.

This is an excellent point and presents an opportunity to better explain the utility and necessity of the MLM. In brief, one challenge of these spatial models is that there are multiple transect points embedded within the three cities that we examined. We could use linear regression to understand how environmental risk is predicted by POE across the 44 transects, and we do so in a supplemental table attached in the supplement, see appendix 6 Global Moran’s I. Unfortunately, this would entail ignoring the inherent dependency in the data where the transects within each site may be influenced differentially across sites. MLM models that site-specific variance but otherwise is akin to regression. Similar to linear regression, there are multiple regression models conducted with a single outcome in order to understand how environmental risk is predicted by each POE. We considered modeling multiple POEs together in a single model so that we could understand intersecting POEs, but the interpretation was complicated by (a) the inherent limitations of the degrees of freedom of 44 transects and (b) lack of independence in each POE from each other. That is, the coefficients from regressions are independent or orthogonal from one another, but many of the POEs are multicollinear and more likely to co-occur than would be expected by chance. It would be helpful to understand how the POEs intersect, but at this nascent stage of the literature it seems prudent to treat each predictor as unique. Backwards elimination, forwards elimination, residualizing and/or interaction terms remain an important and valuable future direction as this field matures.

6. Is it valid to compare different models? Related to previous point, it seems unclear, and based on what were written by the authors, there are more than one models in the study and presented in table 6/figure 4.

We have completely rewritten the results and figures to be responsive to this concern because the reviewer brought up an excellent point. Indeed, this concern prompted us to pay a MLM consultant who is well recognized in the field. Especially with regard to figure 4, the reviewer is accurate that the interpretation of the coefficients in figure 4 were confusing and we now present this data in a way that data points can be compared. Across the different POEs, however, we agree that it is difficult to compare different models directly and indicate this in the figure caption of Figure 4. We considered representing figure 4 using effect sizes and/or standardized betas so that the magnitude of each POE could be compared, but opted against this for two primary reasons: (a) the manuscript currently contains all of the information needed to convert the coefficients to effect sizes under the assumptions of linear regression using, for example, fisher’s r-to-z transformations, and (b) given the practical design-oriented focus of this project, the actual means or intercepts of each POE are more interpretable for the intended audience. If the reviewer would like to see the comparison of effect sizes, however, we would be happy to provide such detail, likely as a supplemental section.

7. Table 6, I would suggest the authors to use ‘*’ to show the statistical significance instead of italicized. Mystery and complexity are not italicized in table 6, are them not significant? Access were italicized, but according to the table footnote, all but access are significant (p<.05). The description in footnote and written in table is conflict.

We concur and now use the conventional. *=p<.05; **=p<.01, and ***=p<.001 nomenclature. Text, footnote, and table have been adjusted to meet this suggestion.

8. Please make sure all relevant data is provided. It seems like the data described in sec 2.3---those data collected with the Fulcrum tool--- is not provided by the authors. According to Plos One policy, all data underlying the findings should be provided without restriction. The data in Table 2 is only showing the mean, SD etc., and the data points behind this info should be provided. The raw data in sec 2.4 seems download-able from publicly available source so it should be fine, but it would be better if a cleaned and extracted copy for only the study sites can also be provided.

All data analyzed in the paper has been cleaned, deidentified, and uploaded as part of this submission.

Reviewer #4: This paper addressed the comments well in the previous version and improved the quality of the paper. Overall, the paper is well-organized, but I still have a few minor comments that need authors to spend more time on that:

1) the introduction is still too long. For section1.1 and 1.2, there is a single paragraph at the beginning, but it is not necessary. These parts are also wordy. Also, the logistic between sections is confusing: why section 1.1 is the literature review? It will be apparent if the authors can make the introduction concise and straightforward.

We totally agree with the comment and decreased the introduction by 20%. We also aimed to make the transition between sections clearer by decreasing compound sentences and breaking apart complex paragraphs that covered more than one idea at a time.

2) The figures are expected in high-quality, especially for the results, but figure 2 and figure 3 are not qualified, missing the axis, title and legend. The texts in Figure 4 are too close and not clear to read. Reviewer 3 also commented on figures 2 and 3 and this oversight has been addressed, thank you. We completely reformatted figure 4 and hope the new figure lends enough conceptual clarity to meet PLoS One’s high quality expectations.

3) The limitations of this paper are not mentioned enough, which also embodied in the data collection and qualitative method.

Another important point and I cleaned up the introduction by emphasizing the subjective nature of the environmental design measures in the limitations.
